# Supplementary material for: Modeling glioblastoma heterogeneity as a dynamic network of cell states
Source: Mol Syst Biol. 2021 Sep 16;17(9):e10105. doi: 10.15252/msb.202010105 (PMC8444284; doi:10.15252/msb.202010105)
Supplement: Supplementary file 6 — Source Data for Figure 5 [file MSB-17-e10105-s004.zip › Figure5A_sourcedata/GSEA_3017/nosigdb_stateF.GseaPreranked.1622795015644/SEURAT_CELLCYCLE.html]

Details for gene set SEURAT\_CELLCYCLE[GSEA]

|  || Dataset | state33017 |
| Phenotype | NoPhenotypeAvailable |
| Upregulated in class | na\_neg |
| GeneSet | SEURAT\_CELLCYCLE |
| Enrichment Score (ES) | -0.80657494 |
| Normalized Enrichment Score (NES) | -4.809909 |
| Nominal p-value | 0.0 |
| FDR q-value | 0.0 |
| FWER p-Value | 0.0 |
Table: GSEA Results Summary

  

Fig 1: Enrichment plot: SEURAT\_CELLCYCLE      
 Profile of the Running ES Score & Positions of GeneSet Members on the Rank Ordered List

  

| PROBE | GENE SYMBOL | GENE\_TITLE | RANK IN GENE LIST | RANK METRIC SCORE | RUNNING ES | CORE ENRICHMENT || 1 | RPA2 |  |  | 1490 | -0.256 | -0.6791 | No |
| 2 | BLM |  |  | 1507 | -0.262 | -0.6820 | No |
| 3 | GAS2L3 |  |  | 1640 | -0.297 | -0.7374 | No |
| 4 | G2E3 |  |  | 1687 | -0.315 | -0.7532 | No |
| 5 | PRIM1 |  |  | 1703 | -0.320 | -0.7546 | No |
| 6 | RAD51 |  |  | 1723 | -0.326 | -0.7577 | No |
| 7 | CDC25C |  |  | 1724 | -0.327 | -0.7521 | No |
| 8 | WDR76 |  |  | 1783 | -0.355 | -0.7727 | No |
| 9 | BRIP1 |  |  | 1790 | -0.357 | -0.7693 | No |
| 10 | NEK2 |  |  | 1815 | -0.371 | -0.7740 | No |
| 11 | CDC45 |  |  | 1836 | -0.382 | -0.7767 | No |
| 12 | E2F8 |  |  | 1864 | -0.396 | -0.7823 | No |
| 13 | POLD3 |  |  | 1918 | -0.436 | -0.7991 | Yes |
| 14 | UHRF1 |  |  | 1921 | -0.438 | -0.7926 | Yes |
| 15 | GINS2 |  |  | 1932 | -0.445 | -0.7895 | Yes |
| 16 | RFC2 |  |  | 1954 | -0.457 | -0.7914 | Yes |
| 17 | NASP |  |  | 1988 | -0.481 | -0.7983 | Yes |
| 18 | CDC6 |  |  | 1991 | -0.483 | -0.7910 | Yes |
| 19 | MCM4 |  |  | 1993 | -0.487 | -0.7831 | Yes |
| 20 | CTCF |  |  | 2010 | -0.502 | -0.7819 | Yes |
| 21 | RANGAP1 |  |  | 2011 | -0.502 | -0.7733 | Yes |
| 22 | FEN1 |  |  | 2018 | -0.511 | -0.7673 | Yes |
| 23 | RRM1 |  |  | 2023 | -0.516 | -0.7603 | Yes |
| 24 | HELLS |  |  | 2038 | -0.528 | -0.7577 | Yes |
| 25 | CDCA3 |  |  | 2041 | -0.529 | -0.7496 | Yes |
| 26 | TTK |  |  | 2047 | -0.533 | -0.7428 | Yes |
| 27 | DTL |  |  | 2066 | -0.573 | -0.7413 | Yes |
| 28 | CENPA |  |  | 2073 | -0.586 | -0.7340 | Yes |
| 29 | AURKB |  |  | 2079 | -0.598 | -0.7261 | Yes |
| 30 | USP1 |  |  | 2084 | -0.612 | -0.7175 | Yes |
| 31 | CKAP5 |  |  | 2086 | -0.623 | -0.7073 | Yes |
| 32 | NCAPD2 |  |  | 2089 | -0.627 | -0.6975 | Yes |
| 33 | CKAP2L |  |  | 2109 | -0.663 | -0.6949 | Yes |
| 34 | AURKA |  |  | 2110 | -0.673 | -0.6834 | Yes |
| 35 | CKS2 |  |  | 2115 | -0.694 | -0.6734 | Yes |
| 36 | CDC20 |  |  | 2117 | -0.695 | -0.6620 | Yes |
| 37 | CDCA2 |  |  | 2120 | -0.705 | -0.6508 | Yes |
| 38 | RAD51AP1 |  |  | 2127 | -0.715 | -0.6414 | Yes |
| 39 | CDCA8 |  |  | 2129 | -0.722 | -0.6295 | Yes |
| 40 | CKAP2 |  |  | 2131 | -0.729 | -0.6175 | Yes |
| 41 | KIF2C |  |  | 2136 | -0.756 | -0.6064 | Yes |
| 42 | NDC80 |  |  | 2142 | -0.775 | -0.5955 | Yes |
| 43 | KIF23 |  |  | 2145 | -0.782 | -0.5830 | Yes |
| 44 | GMNN |  |  | 2146 | -0.785 | -0.5696 | Yes |
| 45 | ANLN |  |  | 2149 | -0.797 | -0.5569 | Yes |
| 46 | TUBB4B |  |  | 2162 | -0.843 | -0.5480 | Yes |
| 47 | ECT2 |  |  | 2163 | -0.846 | -0.5336 | Yes |
| 48 | KIF20B |  |  | 2165 | -0.852 | -0.5195 | Yes |
| 49 | BUB1 |  |  | 2170 | -0.860 | -0.5066 | Yes |
| 50 | LBR |  |  | 2173 | -0.870 | -0.4927 | Yes |
| 51 | NUF2 |  |  | 2176 | -0.877 | -0.4786 | Yes |
| 52 | HMMR |  |  | 2179 | -0.895 | -0.4642 | Yes |
| 53 | HJURP |  |  | 2195 | -0.962 | -0.4547 | Yes |
| 54 | CCNB2 |  |  | 2200 | -0.983 | -0.4397 | Yes |
| 55 | CKS1B |  |  | 2201 | -0.987 | -0.4228 | Yes |
| 56 | KIF11 |  |  | 2206 | -1.008 | -0.4075 | Yes |
| 57 | CENPE |  |  | 2211 | -1.036 | -0.3916 | Yes |
| 58 | CLSPN |  |  | 2216 | -1.060 | -0.3753 | Yes |
| 59 | UBE2C |  |  | 2218 | -1.081 | -0.3573 | Yes |
| 60 | CDK1 |  |  | 2225 | -1.153 | -0.3404 | Yes |
| 61 | ANP32E |  |  | 2227 | -1.200 | -0.3203 | Yes |
| 62 | GTSE1 |  |  | 2230 | -1.220 | -0.3004 | Yes |
| 63 | RRM2 |  |  | 2234 | -1.268 | -0.2801 | Yes |
| 64 | DLGAP5 |  |  | 2235 | -1.273 | -0.2583 | Yes |
| 65 | SMC4 |  |  | 2236 | -1.281 | -0.2365 | Yes |
| 66 | ATAD2 |  |  | 2238 | -1.381 | -0.2133 | Yes |
| 67 | TMPO |  |  | 2239 | -1.382 | -0.1897 | Yes |
| 68 | HMGB2 |  |  | 2241 | -1.469 | -0.1651 | Yes |
| 69 | BIRC5 |  |  | 2242 | -1.469 | -0.1400 | Yes |
| 70 | TOP2A |  |  | 2246 | -1.562 | -0.1147 | Yes |
| 71 | NUSAP1 |  |  | 2247 | -1.600 | -0.0873 | Yes |
| 72 | TPX2 |  |  | 2248 | -1.609 | -0.0598 | Yes |
| 73 | CENPF |  |  | 2249 | -1.691 | -0.0309 | Yes |
| 74 | MKI67 |  |  | 2252 | -1.892 | 0.0005 | Yes |
Table: GSEA details [plain text format]

  

Fig 2: SEURAT\_CELLCYCLE: Random ES distribution      
 Gene set null distribution of ES for **SEURAT\_CELLCYCLE**
